# Direct reprogramming of oligodendrocyte precursor cells into GABAergic inhibitory neurons by a single homeodomain transcription factor Dlx2

**DOI:** 10.1038/s41598-021-82931-9

**Published:** 2021-02-11

**Authors:** Linda L. Boshans, Heun Soh, William M. Wood, Timothy M. Nolan, Ion I. Mandoiu, Yuchio Yanagawa, Anastasios V. Tzingounis, Akiko Nishiyama

**Affiliations:** 1 Department of Physiology and Neurobiology, University of Connecticut, Storrs, CT USA; 2 Department of Computer Science and Engineering, University of Connecticut, Storrs, CT USA; 3 Department of Genetic and Behavioral Neuroscience, Gunma University Graduate School of Medicine, Maebashi, Japan; 4 Institute for Systems Genomics, University of Connecticut, Storrs, CT USA; 5 The Connecticut Institute for Brain and Cognitive Sciences, University of Connecticut, Storrs, CT USA

**Keywords:** Neuroscience, Oligodendrocyte, Differentiation, Developmental biology, Reprogramming

## Abstract

Oligodendrocyte precursor cells (NG2 glia) are uniformly distributed proliferative cells in the mammalian central nervous system and generate myelinating oligodendrocytes throughout life. A subpopulation of OPCs in the neocortex arises from progenitor cells in the embryonic ganglionic eminences that also produce inhibitory neurons. The neuronal fate of some progenitor cells is sealed before birth as they become committed to the oligodendrocyte lineage, marked by sustained expression of the oligodendrocyte transcription factor Olig2, which represses the interneuron transcription factor Dlx2. Here we show that misexpression of Dlx2 alone in postnatal mouse OPCs caused them to switch their fate to GABAergic neurons within 2 days by downregulating Olig2 and upregulating a network of inhibitory neuron transcripts. After two weeks, some OPC-derived neurons generated trains of action potentials and formed clusters of GABAergic synaptic proteins. Our study revealed that the developmental molecular logic can be applied to promote neuronal reprogramming from OPCs.

## Introduction

NG2 glia, also known as oligodendrocyte precursor cells (OPCs or polydendrocytes), represent a fourth major glial population^1^ that self-renew throughout life and proliferate in response to various environmental cues. In the postnatal brain, their physiological fate is largely restricted to the oligodendrocyte lineage^2,3^ except for a small number of neurons in the adult hypothalamus and piriform cortex detected by genetic fate mapping of OPCs, and the production of protoplasmic astrocytes from OPCs in the embryonic ventral gray matter, which ceases after birth (reviewed in^2–4^). A subpopulation of OPCs in the neocortex arises from ventral progenitors in the embryonic medial and caudal ganglionic eminences, which also generate inhibitory neurons^5–9^. Dlx2 is a homeodomain transcription factor that is expressed in ventral telencephalic neural progenitors and is critical for the development of GABAergic neurons. Ventral neural progenitor cells also express the oligodendrocyte basic helix-loop-helix (bHLH) transcription factor Olig2, which is repressed as neural progenitors become committed GABAergic inhibitory neurons by sustained Dlx2 expression^10,11^.

Previous studies have shown that OPCs can be reprogrammed into neurons by a combination of neuronal transcription factors under specific conditions^12–14^. However, the mechanism underlying the fate conversion has not been examined. Our previous bioinformatic study revealed that in OPCs, compared with astrocytes and fibroblasts, the chromatin at key interneuron transcription factor genes is more accessible, with more active and less bivalent or repressive histone posttranslational modifications^15^. In this study, we show that forced expression of a single transcription factor Dlx2 in committed OPCs from the neocortex was sufficient to convert them into immature neurons within two days. Transcriptomic analysis of OPCs two days after Dlx2 transfection revealed upregulation of inhibitory but not excitatory neuronal genes. Subsequently, within 2 weeks Dlx2-transfected OPCs differentiated into purely GABAergic inhibitory neurons, some of which exhibited rapid non-adapting firing patterns and developed inhibitory synaptic puncta.

## Methods

### Animals

The NG2-CreERA BAC transgenic mice (NG2cre)^16,17^ have constitutive cre expression in OPCs and were maintained as a homozygous colony. Gad1-GFP knock-in reporter line (Gad1-GFP)^18^ was generated by inserting EGFP cDNA in-frame into exon 1 of the Gad1 gene and maintained as heterozygotes in a CD1 background. NG2cre mice were crossed to Gad1-GFP to generate heterozygous NG2cre;Gad1-GFP mice. CD1 mice were obtained from Charles River (strain #022; RRID:IMSR_CRL:022). All procedures involving animals were approved by the Institutional Animal Care and Use Committee of the University of Connecticut and carried out in accordance with the NIH and local guidelines and regulations.

### Primary cultures

#### Postnatal cortical OPCs

For culturing postnatal OPCs, we modified the previously published sequential immunopanning purification procedure^19^. We used postnatal day 2–3 (P2–3) CD1 mice from Charles River Laboratory strain #022. After removal of meninges, the cortices were minced and incubated at 35 °C for 60 min in a papain solution (20 U/ml; Worthington). Subsequently, the tissue was triturated in a solution containing ovomucoid and bovine serum albumin (BSA) (Sigma Aldrich) and filtered through a 70-μm cell strainer (Falcon) to obtain a single cell suspension. The cell suspension was incubated in a petri dish coated with the O1 antibody (Gift from Dr. Steven Pfeiffer at University of Connecticut Health Center, 1:1 dilution)^20^ to eliminate oligodendrocytes, with subsequent incubation of the supernatant in a petri dish coated with rat anti-mouse platelet-derived growth factor receptor alpha (PDGFRα) antibody (CD140a, 2 μg/ml, BD Biosciences, RRID:AB_397117) to capture OPCs. Non-adhered cells were washed off with phosphate-buffered saline (PBS), and adhered OPCs were collected by trypsinization. Cells were centrifuged for 5 min at 1100 rpm and re-suspended in DMEM (Thermo Fisher Scientific) supplemented with Sato’s supplements (DMEM-Sato’s) and 50 ng/ml PDGF-AA (Peprotech). Cells were plated on 12-mm glass coverslips (Fisher) coated with 100 μg/ml of poly-d-lysine (Sigma-Aldrich) and 15 μg/ml of laminin (Sigma-Aldrich) or on 35-mm tissue culture dishes (Fisher Scientific) coated with 30 µg/ml of poly-l-lysine (PLL, Sigma-Aldrich). At 3 days post transfection (dpt), cells were trypsinized and re-plated onto a layer of astrocytes at 125,000 cells per 12-mm glass coverslip. Media was switched to neuronal differentiation media consisting of 1% N2 and 1% B27 supplements, 1% l-glutamine, and 1% penicillin and streptomycin in DMEM/F12 (all from Thermo Fisher Scientific) and supplemented with 50 ng/ml human brain-derived neurotrophic factor (BDNF, Peprotech). At 7 dpt, media was completely switched to neuronal maturation media consisting of Neurobasal A (Thermo Fisher Scientific) and supplemented with 2% B27, 1% Glutamax (Thermo Fisher Scientific), and 1% penicillin and streptomycin supplemented with 50 ng/ml human BDNF. Half of media was replaced with fresh media and BDNF every 3–4 days. On 10 dpt, 1% horse serum was added for astrocyte maintenance.

#### Postnatal cortical astrocytes

For culturing primary postnatal astrocytes, we isolated cortical astrocytes as described previously^21^. Briefly, we dissected and chopped the cortices of P1–P3 CD1 mice. Cortical pieces were transferred to a tube containing Hank’s Balanced Salt Solution (Thermo Fisher Scientific) and 0.1% trypsin (Thermo Fisher Scientific) and incubated in 37 °C water bath for 15–30 min. Cells were collected by centrifuging for 5 min at 1100 rpm. Cells were resuspended in astrocyte media consisting of 10% horse serum (Hyclone) and 0.6% d-glucose (Thermo Fisher Scientific) in DMEM) and gently triturated into a single cell suspension. Cells were plated in T75 culture flasks coated with 30 μg/ml PLL. Medium was changed every 2–3 days. At 7–9 days post plating when astrocyte layer was confluent, flasks were shaken at 37 °C at 260 rpm overnight to detach microglia and OPCs. The next day, astrocytes were rinsed with PBS, trypsinized and frozen in astrocyte media with 10% DMSO and 50% horse serum for future use.

### Transfection

Full-length mouse Dlx2 cDNA (NM_010054.2, from translation start site to the stop codon) was cloned into pCMV-IRES2-mCherry expression vector, which had been generated by replacing the EGFP coding region of pIRES2-EGFP vector (Clontech) with mCherry coding region (obtained from Dr. Hitoshi Gotoh, Kyoto Prefectural Medical University). OPCs were transfected 16 h after plating with pCMV-Dlx2-IRES2-mCherry (Dlx2) or control pCMV-IRES2-mCherry (mCherry) plasmid DNA using Lipofectamine 2000 (Invitrogen), and after 5 h, the medium was changed to fresh DMEM-Sato’s medium with 50 μg/ml PDGF-AA.

For utilization of the *Tol2* transposon system to achieve genomic integration of the Dlx2-IRES2-mCherry cassette, Dlx2-IRES2-mCherry was cloned into the cre-dependent *Tol2* transposon donor vector pT2AL-loxP-STOP-loxP-ires2-mCherry vector (kindly provided by Dr. Koichi Kawakami, National Institute of Genetics, Mishima, Japan)^22,23^. OPCs were co-transfected 16 h after plating with Tol2-Dlx2-IRES2-mCherry (Tol2-Dlx2) or Tol2-IRES2-Cherry (Tol2-control) donor plasmids and pCAGGS-T2TP helper plasmid encoding transposase (provided by Dr. Koichi Kawakami) as described above.

### Immunofluorescence labeling

Coverslips were fixed with 4% paraformaldehyde (Electron Microscopy Sciences) in 0.1 M sodium phosphate buffer for 10 min, rinsed three times with PBS and then blocked and permeabilized in PBS containing 0.1% Triton X-100 (Sigma-Aldrich) and 5% normal goat serum (Thermo Fisher Scientific) or 5% normal donkey serum (Thermo Fisher Scientific) at room temperature (RT). The coverslips were then incubated with primary antibodies in blocking solution for 45 min. After three rinses, coverslips were incubated with the secondary antibodies in blocking solution for 30 min. Primary and secondary antibodies are listed in Table 1. Coverslips were mounted in Vectashield mounting medium containing 4′,6-diamidino-2-phenylindole (DAPI) (Vector Laboratories).

**Table 1:** Antibodies used.

Antibody	Source	Cat no.	RRID	Dilution
**Primary antibodies**				
Rat anti-CD140a	BD Biosciences	558774	RRID:AB_397117	1:250
Mouse anti-Tubb3	BioLegend	801202	RRID:AB_10063408	1:3000
Rabbit anti-NG2	Millipore Sigma	AB5320	RRID:AB_91789	1:500
Mouse anti-Olig2	Millipore Sigma	MABN50	RRID:AB_10807410	1:1000
Rabbit anti-Dlx2	Millipore Sigma	AB5726	RRID:AB_2093141	1:1000
Chicken anti-MAP2	Abcam	ab75713	RRID:AB_1310432	1:3000
Mouse anti-Gad67	Millipore Sigma	MAB5406	RRID:AB_2278725	1:3000
Goat anti-Sox2	Santa Cruz Biotechnology	sc-17319	RRID:AB_661259	1:200
Mouse anti-Nestin	Developmental Studies Hybridoma Bank	Rat-401	RRID:AB_2235915	1:8
Goat anti-PDGFRα	R&D systems	AF1062	RRID:AB_2236897	1:1000
Mouse anti-Sox10	Santa Cruz Biotechnology	sc-365692	RRID:AB_10844002	1:1000
Guinea Pig anti-VGAT	Synaptic Systems	131 005	RRID:AB_1106810	1:1000
Mouse anti-PV	Neuromab	L114/3	RRID:AB_2877608	1:100
Rat anti-SST	Millipore Sigma	MAB354	RRID:AB_2255365	1:100
Mouse anti-Gephyrin	Synaptic Systems	147 103	RRID:AB_11041706	1:250
Chicken anti-GFP	Aves Laboratories	GFP1202	RRID:AB_2734732	1:500
Rabbit anti-RFP	Rockland Immunochemicals	KCA379	RRID:AB_2614911	1:1000
O1	Dr. Steven Pfeiffer^a^	N/A	N/A	1:1
**Secondary antibodies**				
Alexa 488-donkey anti-rabbit IgG (H + L)	Jackson ImmunoResearch	711-545-152	RRID:AB_2313584	1:500
Alexa 488-donkey anti-mouse IgG (H + L)	Jackson ImmunoResearch	715-545-150	RRID:AB_2340846	1:500
Alexa 488-donkey anti-chicken IgG (H + L)	Jackson ImmunoResearch	703-546-155	RRID:AB_2340376	1:500
Alexa 488-bovine anti-goat IgG (H + L)	Jackson ImmunoResearch	805-545-180	RRID:AB_2340883	1:500
Alexa 546-donkey anti-mouse IgG (H + L)	Thermo Fisher Scientific	A10036	RRID:AB_2534012	1:500
Alexa 546-donkey anti-rabbit IgG (H + L)	Thermo Fisher Scientific	A10040	RRID:AB_2534016	1:500
Alexa 647-goat anti-rabbit IgG (H + L)	Jackson ImmunoResearch	111-605-144	RRID:AB_2338078	1:200
Alexa 647-goat anti-guinea pig IgG (H + L)	Jackson ImmunoResearch	106-605-003	RRID:AB_2337446	1:200
Alexa 647-goat anti-mouse IgG (H + L)	Jackson ImmunoResearch	115-605-003	RRID:AB_2338902	1:200

^a^University of Connecticut Health Center, Farmington, CT.

### Electrophysiological recordings

Whole-cell patch clamp recordings were performed at room temperature on Dlx2- or control-transfected OPCs grown in culture 14 days after transfection (dpt). Coverslips were scanned to identify mCherry + cells that were GFP + or—using an Olympus BX51WI upright microscope with FITC and TRITC fluorescence and Nomarski optics. Whole-cell recordings were obtained using borosilicate glass electrodes having resistances of 3 to 5 MΩ filled with intracellular solution containing the following (in mM): 130 potassium methylsulfate, 10 KCl, 5 Tris-phosphocreatine, 10 HEPES, 4 NaCl, 4 Mg_2_ATP, and 0.4 Na_4_GTP. The pH was adjusted to 7.2 to 7.3 with KOH. The physiological extracellular solution contained the following (mM), 125 NaCl, 26 NaHCO_3_, 2.5 KCl, 1 NaH_2_PO_4_, 1.3 MgCl_2_, 2.5 CaCl_2_, and 12 glucose and was saturated with 95% O_2_ and 5% CO_2_. Membrane potentials were recorded during 500 ms current injections ranging from -50 to 250 pA in 10 pA increments. Data were acquired through a Multiclamp 700B amplifier (Molecular Devices), low-pass filtered at 10 kHz, and sampled at 50 kHz.

### RNA-sequencing (RNA-seq)

#### RNA extraction, library construction, and sequencing

Immunopurified OPCs were transfected with either control mCherry or Dlx2 plasmid vector. At 2 dpt, transfected cells were isolated by fluorescence-activated cell sorting (FACS) for mCherry with the BD FACSAriaII (Becton Dickinson), using the 488 nm diode laser in the Flow Cytometry Facility (University of Connecticut, Storrs). Three biological replicates consisted of mCherry- and Dlx2-transfected OPCs that had been isolated from different litters of mice. The sorted cells were collected and stored in Trizol reagent (Invitrogen), and total RNA was extracted using the RNeasy Plus Micro Kit (Qiagen). Genomic DNA was removed using gDNA Eliminator spin-columns provided by the kit. RNA concentration and integrity of each sample were measured using an Agilent 2100 Bioanalyzer (Agilent), housed in the Center for Genome Innovation (University of Connecticut, Storrs). Samples with an RNA Integrity Number ≥ 8 were processed. Ribosomal RNA was removed from samples using RiboGone™ (Clontech). cDNA libraries were prepared with 100 ng total input RNA using the SMARTer Stranded RNA-Seq Kit (Clontech). Libraries were multiplexed and subjected to a total of 18 cycles of PCR amplification, and the amplified cDNA library was sequenced at the Center for Genome Innovation (University of Connecticut, Storrs, CT) using Illumina NextSeq 500. Barcoded and multiplexed samples were run on four lanes to generate four technical replicates of 150-nucleotide paired end reads.

#### Data processing, mapping, and differential expression analysis

Illumina adapters were removed, and sample raw reads were trimmed using Trimmomatic v0.39 (RRID:SCR_011848) and filtered based on a quality score ≥ 20. Trimmed sequences were aligned to the mouse reference genome (GRCm38 version 84) using HISAT2 v2.1.0 (RRID:SCR_015530). Output files from HISAT2 were sorted using SAMtools (RRID:SCR_013035), and counts were generated using HTseq-count v0.8.0(RRID:SCR_011867). To obtain normalized counts in fragments per kilobase per million (FPKM), we used TopHat2 v2.1.0 (RRID:SCR_013035) to align the trimmed sequences to the mouse reference genome, and Cufflinks v2.2.1 (RRID_SCR_014597) to estimate transcript abundance. To identify differentially expressed (DE) genes, count tables from HTseq-count were fed into DESeq2. Differential expression analyses were conducted with R v3.2.2(RRID:SCR_001905). Differentially expressed genes with *p*-adj < 0.01 were considered significant.

#### Gene ontology (GO) enrichment analysis

GO enrichment analysis of the differentially expressed genes was performed to identify biological processes enriched in differentially expressed genes using the enrichGO function of clusterProfiler v3.6^24^ (RRID:SCR_016884). The GO over-representation test^25^ was performed using the Benjamini & Hochberg Method, with the cut-off values for *p* and *q* set to 0.01 and 0.05, respectively.

### Quantitative PCR

Immunopurified OPCs were transfected with either control mCherry or Dlx2 plasmid vector, with a transfection efficiency of 10–15%, and cultured as described above. The entire cell population, including transfected and un-transfected, was lysed at 2, 7, and 14 dpt, and total RNA was purified with the PureLink RNA Mini Kit (Thermo Fisher Scientific) according to manufacturer’s protocol. RNA was reverse transcribed into cDNA using Superscript IV First-Strand Synthesis System (Invitrogen) with a mixture of random primers and oligo(dT) primers (Invitrogen), according to manufacturer’s protocol. SsoAdvanced Universal SYBR Green Supermix (BioRad) was used to perform qPCR in a BioRad CFX96 Touch Real-time PCR Detection System with 20 ng of cDNA and the primers listed in Table 2A,B. Three experimental replicates were used per condition.

**Table 2:** Primers used for qPCR.

Primer name	Primer orientation	Primer sequence
**(A) Dlx genes for Fig.** 3B-F		
Dlx1	Forward	5'-CTACAGTTCGGCCTCATCCTTC-3'
	Reverse	5'-TTCCACCACCGTACTCTTCTCG-3'
Dlx1as	Forward	5'-GCAGACAGAATTGGGTCGTT-3'
	Reverse	5'-CTCAACTACCGCCTGCAAA-3'
Dlx2	Forward	5'-TCCTACTCCGCCAAAAGCAG-3'
	Reverse	5'-GAGACGAACTGGTGCCGTAG-3'
Dlx5	Forward	5'-GTTTGACAGAAGAGTCCCAAGC-3'
	Reverse	5'-TTGGCGATTCCTGAGACGG-3'
Dlx6	Forward	5'-GTGGGTTACTACCCTGCTTCA-3'
	Reverse	5'-GACTCAATACCTGGCCCTTCC-3'
Dlx6as (Evf2)	Forward	5'-CAAACAGGGGATGGGGTTCA-3'
	Reverse	5'-TGCCTACAGTCGCATAGCTC-3'
**(B) Inhibitory neuron genes for Fig.** 4G		
Ccnd2	Forward	5′-CTTCTTCGGGGAGAACCACC-3'
	Reverse	5′-CTTCTGTCCCAGGGCAAGTC-3'
Epha5	Forward	5′-TGGAGAGAGACCCTACTGGGA-3'
	Reverse	5′-TGATAGAGAGCAGCAGGGCA-3'
Erbb4	Forward	5′-GCTGCTGTTGAACTGGTGTG-3'
	Reverse	5′-TCACTAAGACATTGCGGGCT-3'
Gad1	Forward	5′-GAGCGATCAAATGTCTTGCGG-3'
	Reverse	5′-ACAGAGACCGACTTCTCCAAC-3'
Mef2c	Forward	5′-CACGAGAGCCGGACAAACT-3'
	Reverse	5′-AGGTGGAACAGCACACAATCT-3'
Plcxd3	Forward	5′-GCAAACACGACAGACCCAGA-3'
	Reverse	5′-TGCATCATGGCGGGAAGAG-3'
Pvalb	Forward	5′-GGCCTGAAGAAAAAGAACCCG-3'
	Reverse	5′-ATCTTGCCGTCCCCATCCTT-3'
Satb1	Forward	5′-GTGCGGGATGAACTGAAACG-3'
	Reverse	5′-GGCTTCCGGCAACTGTAAGA-3'
Sst	Forward	5′-CCCCAGACTCCGTCAGTTTC-3'
	Reverse	5′-GGCTCCAGGGCATCATTCTC-3'
St18	Forward	5′-CAGGGCAAGGACAAAAAGCAC-3'
	Reverse	5′-AGCGGGTGGAAAGGTTCAG-3'
Vip	Forward	5′-TAGCAGAAAATGGCACACCCTA-3'
	Reverse	5′-TGTCGTTTGATTGGCACAGG-3'

### Data analysis and statistical evaluation

Images of fluorescently labeled dissociated cells were acquired using a Leica SP8 confocal microscope (Advanced Light Microscopy Facility, University of Connecticut, Storrs) or Zeiss Axiovert 200 M. For quantification, images were captured from random fields on coverslips from a minimum of three separate experiments (8–16 male and female mouse pups per experiment) and immunolabeled cells in each field were counted. Quantification data are represented as means ± standard deviations. For RNA-seq, three biological replicates, consisting of 2 or more immunopanning experiments per replicate, were used per condition. For electrophysiological recordings, neurons were recorded from two separate experiments. Generation of graphs and statistical analysis of data was performed with GraphPad Prism (Version 7 and 8) software or R (v3.2.2, RRID:SCR_001905). Images were processed and analyzed using ImageJ (RRID:SCR_001935), and Adobe Photoshop (RRID:SCR_014199) was used to generate figures.

## Results

### Differentiation of Dlx2-transfected postnatal OPCs into GABAergic neuron-like cells

**Figure 1:**
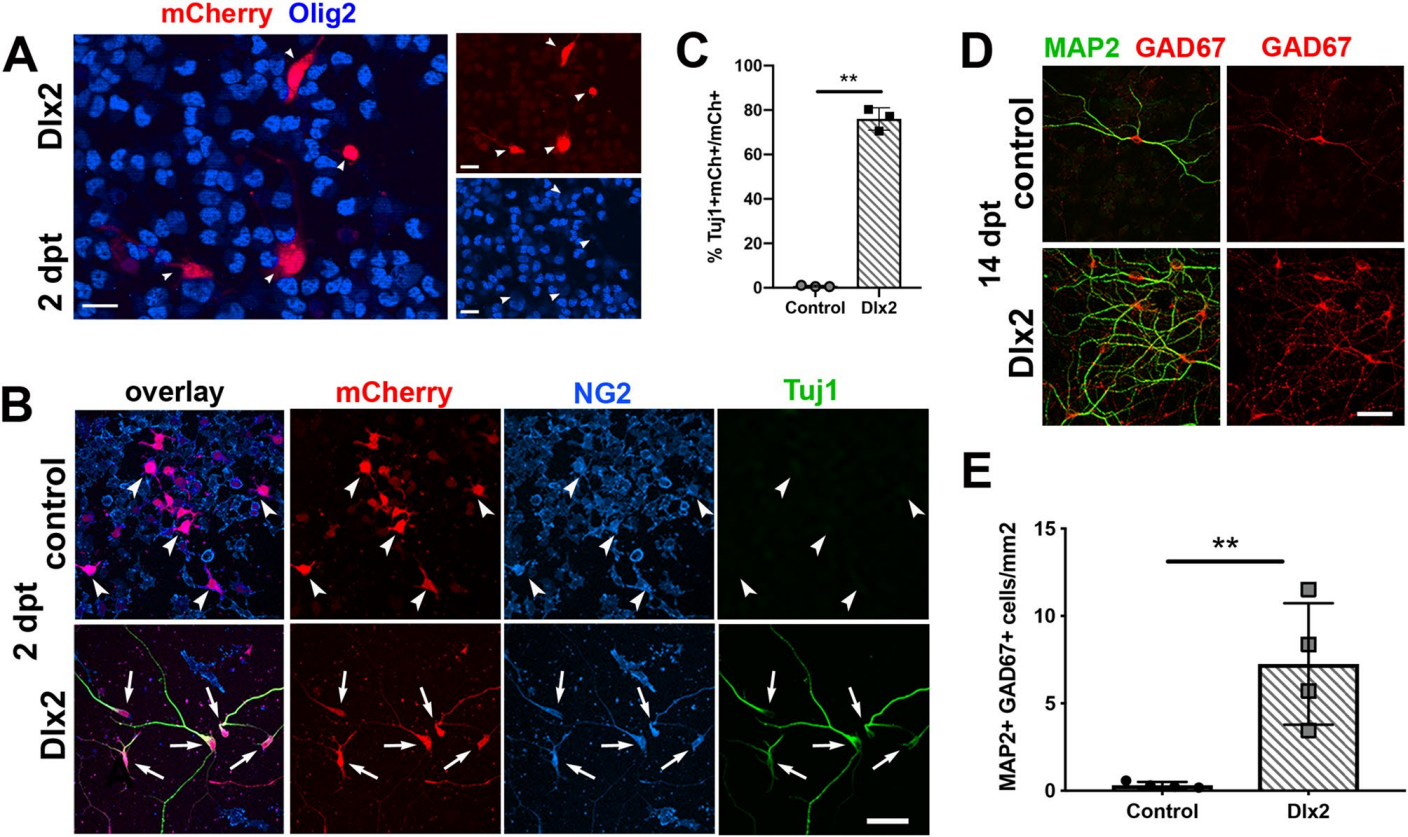
Dlx2 misexpression converts OPCs into immature GABAergic neurons. (**A)** Dlx2-transfected OPCs immunolabeled 2 days post transfection (dpt) for mCherry (red) and Olig2 (blue). Scale bars, 20 µm. (**B)** Control-transfected (top) and Dlx2-transfected (bottom) OPCs immunolabeled at 2 dpt for Tuj1 (green), mCherry (red) and NG2 (blue). Arrowheads in the top control-transfected panels indicate mCherry + cells that are NG2 + but Tuj1-negative. Arrows in the bottom Dlx2-transfected panels indicate mCherry + cells that express both Tuj1 and NG2. Scale bar, 50 µm. (**C)** Bar graph showing the proportion of transfected cells that became Tuj1 + neurons in control- and Dlx2-transfected OPCs. Means ± standard deviations, *n* = 3, ** *p* = 0.0014. Student’s *t*-test, unpaired. (**D**) Representative examples of neuronal density in control- and Dlx2-transfected OPCs at 14 dpt, immunolabeled for MAP2 (green) and GAD67 (red). Scale bar, 50 µm. (**E**) Density of GAD67 + MAP2 + cells in control- and Dlx2-transfected cultures. Means ± standard deviations, *n* = 4, ** *p* = 0.0073, Student’s *t*-test, unpaired.

Dlx2 represses Olig2 during interneuron development and is not detected in OPCs^10,26^. Previously we showed that OPCs in the postnatal SVZ or the forebrain parenchyma do not express detectable levels of the key interneuron homeodomain transcription factor Dlx2^26^. To examine whether Dlx2 could alter the fate of OPCs that had been committed to the oligodendrocyte lineage, we transfected immunopurified OPCs from postnatal mouse cortex with either control mCherry (control) or Dlx2-mCherry (Dlx2) plasmid. One day after transfection (1 dpt), Dlx2-immunoreactivity was detected in Dlx2- but not in control-transfected OPCs (Supplemental Fig. 1A). As early as 2 dpt, 24.3 ± 4.9% of Dlx2-transfected cells had barely detectable Olig2 (Fig. 1A, arrowheads; Supplemental Fig. 2B) and had larger cell bodies and longer processes (see bottom left mCherry + cell in Fig. 1A and arrows in Fig. 1B), while the remaining Dlx2-transfected cells exhibited weak Olig2 immunoreactivity. This was not due to transfection of Dlx2 into Olig2-negative non-OL lineage cells, since at 1 dpt > 97% of mCherry + cells expressed NG2 and Olig2 in both control- and Dlx2-transfected cultures (Supplemental Fig. 1B). Using other oligodendrocyte lineage markers, we found that at 2 dpt, 45.4 ± 8.6% and 55.6 ± 11.6% of Dlx2-transfected cells showed no detectable immunoreactivity for PDGFRα and Sox10, respectively (Supplemental Fig. 2A–D). Furthermore, by 2 dpt, 76.0 ± 5.0% of mCherry + Dlx2-transfected cells expressed the neuronal cytoskeletal protein βIII-tubulin (Tuj1), while they continued to express the OPC antigen NG2 (Fig. 1B,C, arrows). By 7 dpt, 73.3 ± 6.6% and 76.9 ± 11.1% were negative for PDGFRα and Sox10, respectively, (Supplemental Fig. 2E–H), indicating that Dlx2-transfected cells had exited from the oligodendrocyte lineage. Thus, overexpression of Dlx2 in postnatal OPCs had caused them to switch their fate from oligodendrocytes to immature neurons.

**Figure 2:**
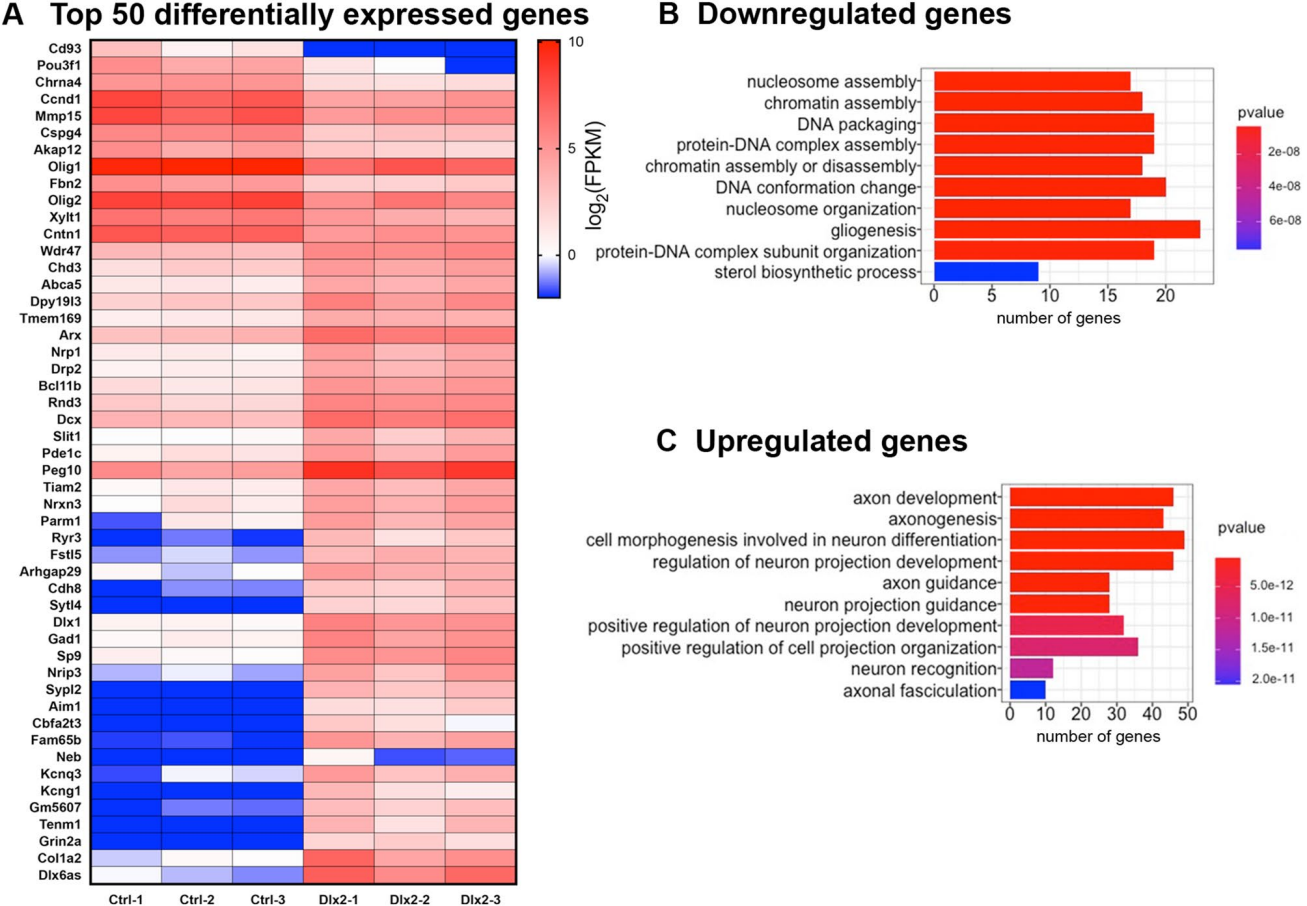
Dlx2 induces GABAergic neuronal genes in OPCs within 2 days. (**A)** Heatmap of the 50 most significantly differentially expressed genes. Columns represent control-transfected biological replicates (Ctrl-1, Ctrl-2, Ctrl-3) and Dlx2-transfected biological replicates (Dlx2-1, Dlx2-2, Dlx2-3). Color scale depicts log_2_(FPKM) values. (**B,C**) The top 10 most significant gene ontology (GO) terms enriched for significantly upregulated (**B**) or significantly downregulated (**C**) genes, graphed in order of significance. Horizontal-axes represent the number of genes matched to each GO term. Legends show the color scale for the *p*-values.

By 14 dpt, Dlx2-transfected OPCs expressed microtubule-associated protein 2 (MAP2), known to be present in neuronal soma and dendrites, and glutamic acid decarboxylase (GAD67), which converts glutamate into GABA in inhibitory GABAergic neurons. In Dlx2-transfected cultures, there were clusters of MAP2 + GAD67 + cells, which were 30-fold more abundant than in control-transfected cultures (Fig. 1D-E), and 99.4 ± 0.4% of the MAP2 + cells expressed GAD67. There were occasional isolated GAD67 + MAP2 + cells in control-transfected cultures. These were likely to have arisen from contaminating neuroblasts in the subventricular zone (SVZ), which were present in up to 2% of the immunopurified OPCs. Consistently, at 2 dpt, 0.7% ± 0.3% of the mCherry + cells in control-transfected cultures expressed Tuj1 (Fig. 1C). These observations suggest that forced expression of Dlx2 in postnatal oligodendrocyte lineage-committed cells led them to switch their fate toward GABAergic inhibitory neurons.

### Dlx2 induces GABAergic neuronal genes in OPCs within 2 days

To explore the molecular mechanism underlying the fate switch from OPC to GABAergic neurons by Dlx2, we compared transcriptomic profiles of control- and Dlx2-transfected OPCs after 2 dpt. Among the 740 significantly differentially expressed genes (*p-*adj < 0.01), 445 were upregulated and 295 were downregulated in Dlx2-transfected cells (Supplementary Table 1). Figure 2A shows a heatmap of the 50 most significantly differentially expressed genes ranked by *p*-adj values. Among the most significantly downregulated genes were Cspg4, encoding NG2, and the oligodendrocyte transcription factors Olig1 and Olig2. Gene ontology (GO) term analysis revealed that gliogenesis was the largest represented biological process with the largest number of downregulated genes (Fig. 2B). Other significantly downregulated genes in this GO category were Pdgfra (ranked 209 by *p*-adj) and Sox10 (ranked 705). Other biological processes enriched in downregulated genes were related to chromatin assembly and organization, with the majority of the genes in these groups encoding histone proteins, suggesting changes in the chromatin structure during neuronal reprogramming of OPCs.

Among the significantly upregulated genes were those known to be expressed by immature neurons such as Dcx (Doublecortin) and Tubb3 (encoding βIII-tubulin) (Fig. 2A, Supplementary Table1), as well as other genes important for neuronal function such as those encoding voltage-dependent K^+^ channels (Kcnq3 and Kcng1) and synaptic proteins such as Grin2a (NMDA receptor 2A) and Sytl4 (synaptotagmin-like protein 4). Among the most significantly upregulated transcription factors was Sp9, which regulates the expression of Fgf8 (fibroblast growth factor 8) implicated in telencephalic patterning^27^. GO analysis of the upregulated genes revealed an enrichment of genes that are important for neurogenesis and neuronal differentiation (Fig. 2C). Nine out of ten GO terms that were enriched in upregulated genes in Dlx2-transfected cells were related to axonal growth.

We also examined genes that regulate the cell cycle and are known to be expressed in OPCs^28,29^. The majority of the positive regulators of the cell cycle were downregulated in Dlx2-transfected cells compared to control-transfected cells (Supplemental Fig. 3A), with Ccnd1, encoding the G1/S-specific cyclin-D1, being the most strongly downregulated. This suggests that Dlx2-transfected OPCs had become post-mitotic as they differentiated into inhibitory neurons, consistent with previous reports that neuronal differentiation from neural progenitor cells is initiated through downregulation of the activity of cyclins and CDK proteins^30,31^.

**Figure 3:**
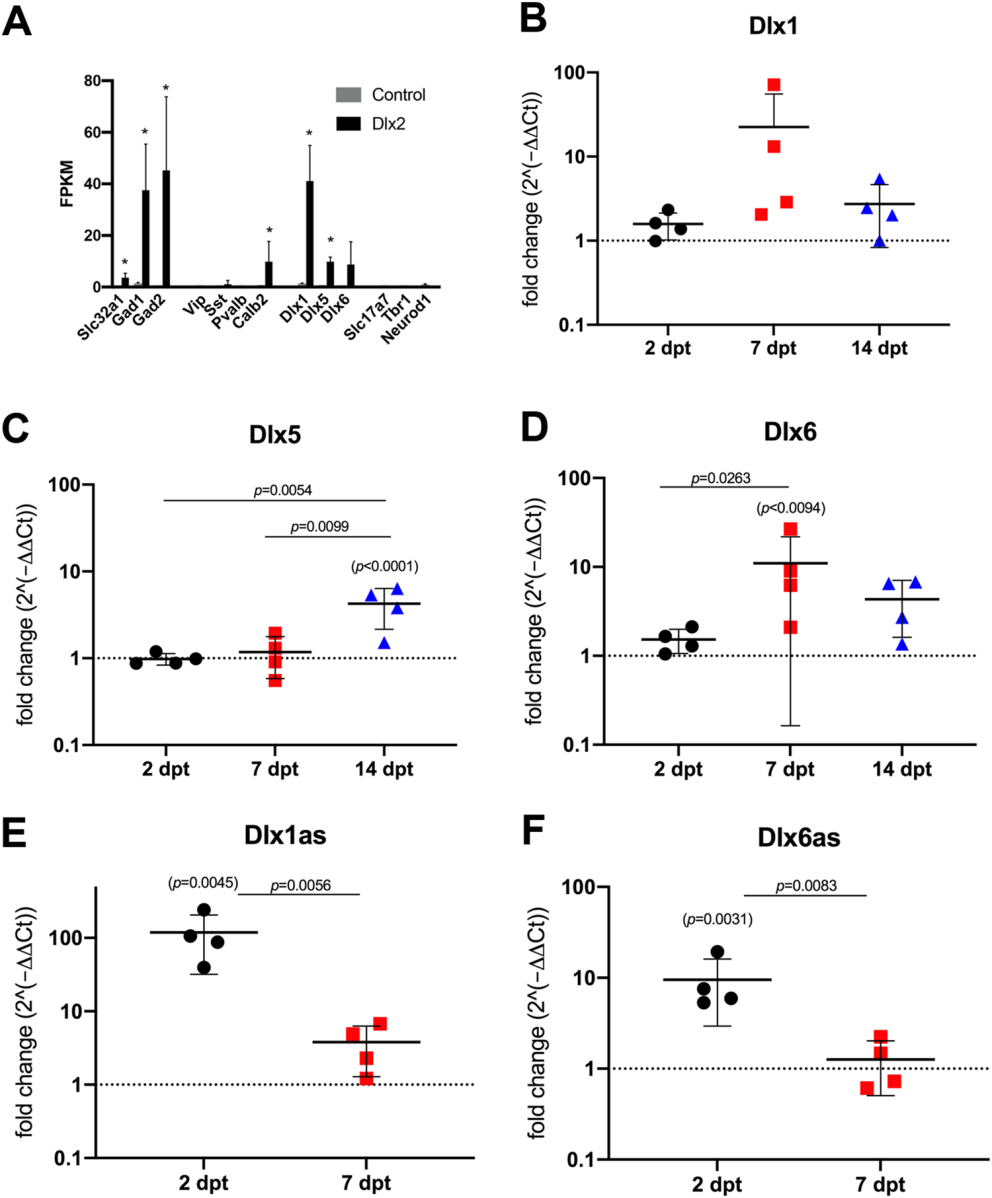
Dynamic changes in neuronal transcripts in Dlx2-transfected OPCs. (**A)** FPKM values of key neuronal transcripts in control- and Dlx2- transfected OPCs. The *p-adj* values for the transcripts are as follows: Gad1 1.64 × 10^–19^, Gad2 1.21 × 10^–9^, Slc32a1 0.0001761, Dlx1 1.34 × 10^–23^, Dlx5 0.0007, Calb2 6.80 × 10^–10^. * denotes *p-adj* < 0.05. *n* = 3. (**B–F)** The expression of Dlx1 (**B**), Dlx5 (**C**), Dlx6 (**D**), Dlx1as (**E**) and Dlx6as (**F**) transcripts at 2, 7, and 14 dpt obtained by qPCR. Values are for Dlx2-transfected OPCs and are normalized to control-transfected OPCs (black dotted line), and shown as fold-change over control-transfected OPCs in log_10_ scale. The *p*-values for the comparison between Dlx2-transfected cells and control-transfected cells are indicated above each dataset in parentheses. The *p*-values for the comparison of Dlx2-transfected cells at 2, 7, and 14 dpt are indicated above the horizontal bars. Two-way ANOVA, Sidak’s multiple comparisons test, *n* = 4.

#### Upregulation of GABAergic but not glutamatergic neuronal genes

We next further examined neuronal genes that were upregulated in Dlx2-transfected OPCs (Fig. 3A; Supplementary Table 1). There was a significant upregulation of Gad1 and its closely related gene Gad2, which encode GAD67 and GAD65, respectively, in GABAergic inhibitory neurons^32,33^, as well as upregulation of Slc32a1, which encodes vesicular GABA transporter (VGAT). However, we did not detect transcripts expressed by the major non-overlapping mature interneuron classes in the neocortex, Sst encoding somatostatin (SST), Pvalb encoding parvalbumin (PV), and Vip encoding vasoactive intestinal peptide (VIP), while we saw an upregulation of Calb2 encoding calretinin, also known as calbindin-2, expressed by a subset of VIP + and SST + interneurons^34^. Within the Dlx family of interneuron transcription factors, Dlx1, and Dlx5, to a lesser extent, were upregulated. By contrast, we did not detect the excitatory neuron bHLH transcription factor Neurod1^13^ in Dlx2- or control-transfected OPCs. Nor did we see upregulation of other key genes expressed by cortical excitatory neurons such as Slc17A7 encoding vesicular glutamate transporter VGLUT1^35^ or Tbr1 (T-box brain protein 1) encoding a transcription factor expressed in pyramidal neurons in the deep neocortical layers (Fig. 3A)^36^. These findings indicate that transient episomal Dlx2 misexpression in OPCs was sufficient to selectively upregulate inhibitory neuron transcription factor expression and initiate the program of differentiation towards GABAergic inhibitory neurons in 2 days.

#### Changes in chromatin modifiers during fate switch into GABAergic neurons

To predict potential changes to the chromatin architecture during the early phase of neuronal reprogramming of OPCs, we next examined the RNA-seq dataset at 2 dpt to see whether Dlx2 overexpression altered the levels of transcripts encoding chromatin modifying factors. Among histone deacetylases (HDACs), there was a 3.7-fold upregulation of Hdac11 (Supplemental Fig. 3B), which encodes a class IV histone deacetylase and is implicated in spinal motor neuron survival by interacting with the mRNA splicing machinery^37^. Notably, there was no statistically significant change in Ezh2, which encodes the catalytic subunit of the polycomb repressive complex 2 (PRC2) responsible for depositing the repressive trimethylation mark on histone 3 lysine 27 (H3K27) at many inhibitory neuron genes in OPCs^15,38,39^. Nor was there any change in expression of Kdm6b, which encodes H3K27 demethylase. Among other histone posttranslational modifiers, Kdm3a and 7a were significantly upregulated. Kdm3a demethylates mono- and di-methyl H3K9. It interacts with Neurogenin-2 and increases chromatin accessibility for NeuroD1 and Tubb2b during neuronal differentiation^40^. Kdm7a, which demethylates di-methyl H3K27 and H3K9, promotes neural differentiation by increasing FGF4 expression^41^. Interestingly, among the transcripts encoding ATP-dependent chromatin remodeling enzymes, chromodomain helicase DNA binding protein 3 (Chd3), implicated in cortical layer specification^42^, was significantly upregulated. DNA methylation also regulates chromatin structure and gene transcription through the deposition of methyl groups at CpG islands. Dnmt1, which encodes maintenance DNA methyltransferase essential for correct alternative splicing during OL differentiation^43^, was significantly downregulated in Dlx2-transfected OPCs (Supplemental Fig. 3C). By contrast, the level of Dnmt3a encoding de novo DNA methyltransferase was not changed in Dlx2-transfected OPCs. These changes triggered by Dlx2 overexpression could have caused nucleosome repositioning and facilitated activation or derepression of critical genes during differentiation of OPCs into GABAergic neurons.

### Dynamic changes in Dlx gene expression during neuronal reprogramming from OPCs

To explore the mechanism of neuronal reprogramming from OPCs, we used qPCR to examine the temporal changes in transcription factor transcripts known to be critical for interneuron development in Dlx2-transfected cultures. We focused on other members of the Dlx family transcription factors, since they were upregulated in our initial RNA-seq analysis at 2 dpt and are known to be influenced by Dlx2. During development, Dlx2 is expressed early and transiently and directly activates Dlx5 and 6, which are critical for inhibitory neuron differentiation and maturation^44^. Dlx1 expression is co-regulated with Dlx2^45^. Its expression is transient in PV + interneurons but sustained in SST + interneurons. In Dlx2-transfected OPCs, Dlx1 expression peaked at 7 dpt, although it did not reach significance, likely due to large variability among samples. By 14 dpt, Dlx1 returned to a level only slightly above control (Fig. 3B). As predicted from developmental expression, the upregulation of Dlx5 expression in Dlx2-transfected OPCs was most prominent at14 dpt, although a small increase was observed by RNA-seq at 2 dpt (Fig. 3C). Dlx6 expression increased earlier than Dlx5, with peak expression at 7 dpt, and decreased but remained slightly elevated at 14 dpt (Fig. 3D).

Interestingly, two long non-coding RNAs (lncRNAs) in the Dlx family were highly upregulated (Fig. 3E-F and Supplementary Table 1). Dlx1as, which is an antisense lncRNA to Dlx1, has been shown to modulate Dlx1 expression^46,47^. Dlx6as, an antisense lncRNA to Dlx6, also known as Dlx6os1 or Evf2, has been shown to promote neurogenesis by inhibiting DNA methylation at the ultraconserved enhancer of Dlx5/6^48^. At 2 dpt, we detected a > 100-fold upregulation of Dlx1as. Dlx6as transcript was also elevated early at 2 dpt. Both transcripts declined by 7 dpt.

### Functional and morphological differentiation of OPC-derived GABAergic neurons

#### OPC-derived immature neurons generate action potentials

We noticed that episomal mCherry expression disappeared beyond the first week after transfection. To achieve persistent reporter expression in OPC-derived neurons, we transfected OPCs from NG2cre;Gad1-GFP mice^16–18^ with a cre-dependent *Tol2* transposon donor vector Tol2-Dlx2-ires2-mCherry (Tol2-Dlx2) or control Tol2-ires2-mCherry (Tol2-control) and the T2 transposase helper vector. This enabled identification of GABAergic neurons by GFP and Dlx2-transfected cells by mCherry expression. We detected Gad1-GFP in neuron-like cells as early as 3 dpt in Tol2-Dlx2-transfected cells, consistent with the early upregulation of Gad1 mRNA from our RNA-seq data. At 14 dpt, 93.6 ± 0.4% of mCherry + cells were also Gad1-GFP + in Tol2-Dlx2-transfected OPCs compared to 1.2 ± 1.1% in Tol2-control-transfected OPCs. Most of the latter remained in the oligodendrocyte lineage and expressed Olig2 (Supplemental Fig. 4A, arrowheads). The small number of Gad1-GFP + cells found in Tol2-control-transfected cultures were mCherry-negative (Supplemental Fig. 4B), also supporting the notion that they were likely to have arisen from untransfected neuroblasts in the original OPC culture.

**Figure 4:**
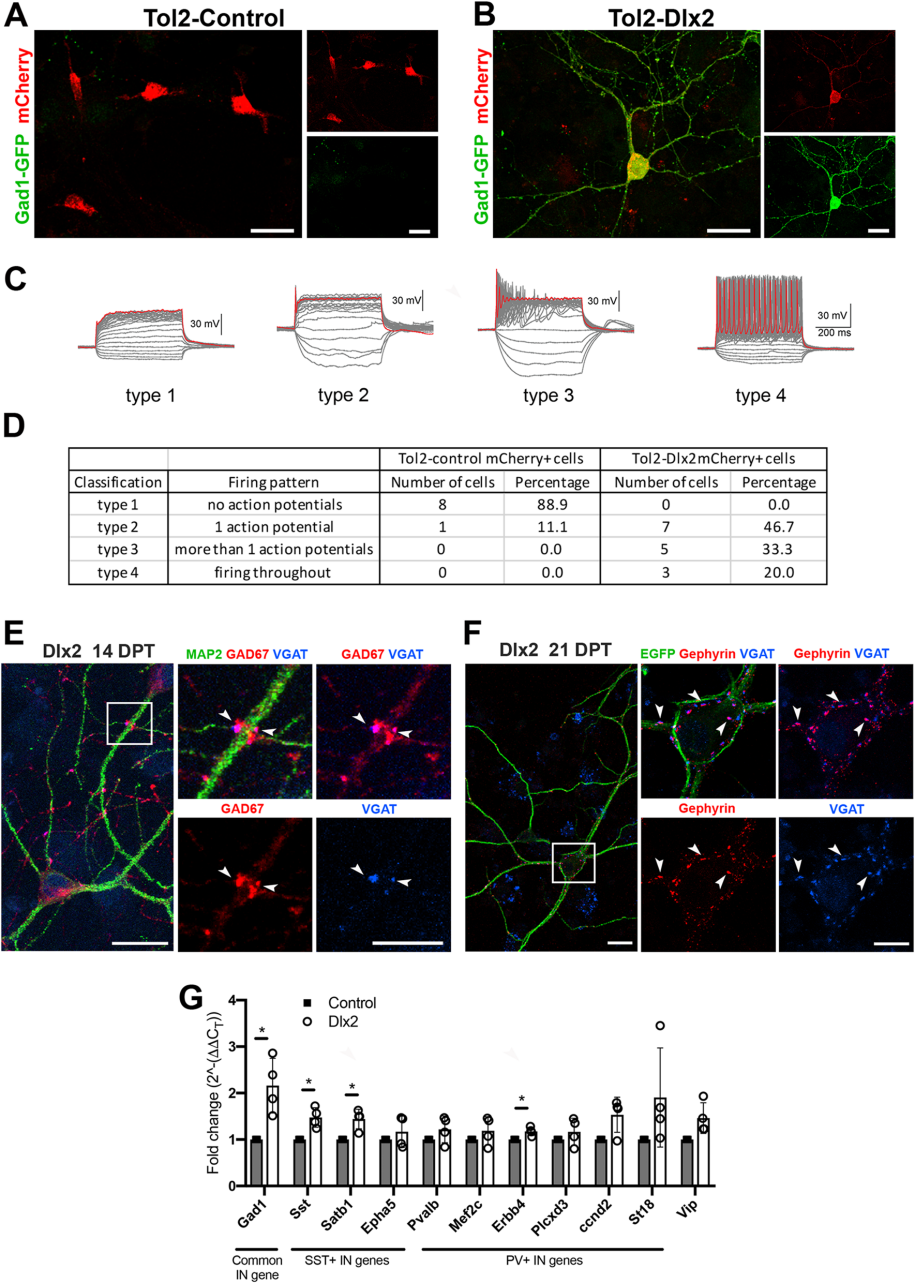
Differentiation of OPC-derived GABAergic neurons. (**A,B)** Representative mCherry + cells that were recorded from in Tol2-control-transfected (**A**) and Tol2-Dlx2-transfected (**B**) OPCs at 14 dpt expressing Gad1-GFP (green) and mCherry (red). Scale bar, 30 µm. (**C,D)** Membrane voltage traces in response to current injection (− 50 to + 250 pA, 500 ms duration, 10 pA increments). Red indicates response to highest current injection. Recorded cells are categorized into four types, based on the electrical response to voltage clamp recordings (**C**), and listed in detail in table (**D**). (**E)** Co-localization of GAD67 (red) and VGAT (blue) at 14 dpt in Dlx2-transfected OPCs. Right panels show higher resolution images of the boxed region in left panel. Arrowheads indicate area where GAD67 and VGAT double positive puncta wrap around MAP2 + (green) dendrites. Scale bars, 20 µm (low resolution image) and 10 µm (higher resolution images). (**F)** Co-localization of inhibitory post-synaptic protein gephyrin (red) and pre-synaptic inhibitory protein VGAT (blue) at 21 dpt in Dlx2-transfected OPCs. Right panels show higher resolution images of the boxed region in left panel. Arrowheads indicate synapsetic puncta with colocalized VGAT and gephyrin. Scale bars, 20 µm (low resolution image) and 10 µm (higher resolution images). (**G)** Expression of inhibitory neuron subtype-specific genes at 14 dpt for control- or Dlx2-transfected OPCs analyzed by qPCR. Values are normalized to the average of control-transfected cells for each transcript. The *p-*values for each transcript are as follows: Sst 0.0200, Satb1 0.0247, Erbb4 0.0305. Asterisk denotes *p-*values < 0.05. n = 4, Student’s *t*-test, unpaired.

Using the optimized method of sustaining reporter expression in Dlx2-transfected cells, we examined the excitability of mCherry + OPC-derived GABAergic neurons at 14 dpt using whole cell patch-clamp electrophysiological recordings. In current-clamp configuration, we examined whether cells could generate action potentials in response to a series of depolarizing current injections. When we recorded from Tol2-control-transfected mCherry + cells, we saw a graded voltage response but no action potentials in 8 out of 9 recorded cells (Fig. 4A,C,D). We categorized cells with this pattern of voltage change as type 1 cells. One cell in the control cultures exhibited a single action potential (categorized as a type 2 cell). We were unable to record from GFP + mCherry + cells in control OPCs as they were extremely rare and difficult to locate. When we examined mCherry + Gad1-GFP + cells in Tol2-Dlx2-transfected cultures, we observed a range of different types of responses (Fig. 4B–D). About half of the cells exhibited a single action potential (type 2 cells), and one-third of the cells displayed a burst of several action potentials that quickly adapted in the continuous presence of depolarizing current (type 3 cells), indicative of immature neurons. Notably, in 3 out of 15 cells we detected continuous non-adapting spiking trains of action potentials (type 4 cells). The heterogeneous action potential patterns likely reflect the transitional state of OPC-derived neurons from an electrically immature to a more mature state at 14 dpt after Dlx2 misexpression.

#### OPC-derived immature neurons develop GABAergic synaptic puncta

To look for morphological evidence of synapse formation in OPC-derived neurons, we immunolabeled for synaptic proteins. At 14 dpt, we observed clustering of VGAT at the junction between a GAD67 + axon and a MAP2 + GAD67 + dendrite in areas of high cellular density (Fig. 4E arrowheads), indicative of an early stage of synapse formation. At 21 dpt, we saw clusters of VGAT and the inhibitory post-synaptic scaffolding protein gephyrin around the soma and proximal process of MAP2 + neurons derived from Dlx2-transfected OPCs, indicating the formation of morphological synapses (Fig. 4F, arrowheads). By contrast, none of the neurons in control-transfected cultures expressed VGAT. In Dlx2-transfected cultures we did not detect synaptic puncta that contained VGLUT1 or 2 and the excitatory post-synaptic scaffolding protein PSD-95, consistent with the lack of excitatory neuronal transcripts detected by the RNA-seq. Thus, OPC-derived GABAergic neurons formed exclusively inhibitory morphological synapses amongst themselves.

### OPC-derived interneurons acquire a mixed neurochemical phenotype

We next examined whether OPC-derived GABAergic neurons were differentiating into a specific class of inhibitory neurons by examining the presence of transcripts known to be expressed by PV + and SST + neurons^36,49^. We focused our analysis on signature genes of PV + and SST + interneuron classes, as they make up the majority of the cortical inhibitory neurons. We performed qPCR on Dlx2-transfected and control-transfected OPCs at 14 dpt to determine whether any of the transcripts were upregulated in Dlx2-transfected OPCs. For the genes known to be expressed by PV + neurons, we assessed, in addition to Pvalb, (1) Mef2c, a transcription factor critical for PV + interneuron development; (2) Erbb4, a receptor tyrosine kinase (RTK) that binds epidermal growth factor; and (3) Plcxd3, a phospholipase C protein^50^. We also included early markers of PV + interneurons: Ccnd2 encoding the G1/S cyclin D2 and St18, a transcription factor involved in interneuron migration^51^. Of these, only Erbb4 was significantly upregulated in Dlx2-transfected cells compared to control-transfected cells, although there was a trend for increased expression of the other genes in Dlx2-transfected (Fig. 4G).

We next examined genes that are known to be expressed by SST + interneurons^36,49^. At 14 dpt Dlx2-transfected cultures expressed a significantly higher level of mRNA encoding Sst and Satb1, a transcription factor that plays a critical role in the specification and maturation of SST + interneurons and persists in adult SST + interneurons^52,53^. We did not detect a significant change in Epha5, an early marker of SST + interneurons that disappears upon maturation^51^. Vip expression was not significantly upregulated. As expected, Gad1 expression, which is common to all interneuron classes, was significantly upregulated in Dlx2-transfected OPCs. To examine whether Dlx2-transfected OPCs expressed the signature interneuron proteins, we immunolabeled 14 dpt OPC-derived neurons for SST or PV. However, we did not observe any immunoreactive cells, despite strong immunolabeling with both antibodies on tissue sections (not shown). These data suggest that OPC-derived interneurons have not yet matured into an unambiguous class of interneurons. This could be due to class heterogeneity among OPC-derived neurons or that they were differentiating into a cell type with intermediate properties of SST + and PV + neurons.

## Discussion

In this study, we show that misexpression of a single transcription factor Dlx2 caused postnatal neocortical OPCs to switch their fate from oligodendrocyte lineage to GABAergic inhibitory neurons. RNA-seq analysis of OPCs during the window of fate conversion into neurons revealed that the transcriptional program towards inhibitory neuron differentiation had already begun within two days of exposure to Dlx2. OPC-derived neurons exhibited clustering of GABAergic synaptic proteins, and the active membrane properties of some resembled those of PV + inhibitory neurons at 14 dpt. However, transcriptional analysis at 14 dpt did not reveal evidence for full maturation into a specific class of inhibitory neurons.

### Intrinsic properties that facilitate reprogramming of OPCs into GABAergic neurons

Previous reports that used Dlx2 to reprogram non-neuronal cells into inhibitory neurons invariably required additional transcription factors, particularly Ascl1, which functions as a “pioneer” transcription factor. Pioneer factors alter the chromatin landscape around closed chromatin regions by recruiting chromatin remodeling complexes and enzymes that catalyze histone posttranslational modifications, thereby making the key neuronal genes accessible to neurogenic transcription factors^54^. The presence of Ascl1 has been shown to increase the efficiency of Dlx2 to reprogram astrocytes into GABAergic interneurons from 35 to 93%^55^. By contrast, our results showed that Dlx2 alone could reprogram OPCs into inhibitory neurons at an efficiency of 94%. One possible explanation for the ability of Dlx2 alone to convert OPCs into inhibitory neurons without other transcription factors is that OPCs express a low level of Ascl1 (Supplementary Table 1)^56,57^, obviating the need for exogenous Ascl1. This is supported by our recent finding that in OPCs genes encoding the key critical transcription factors for inhibitory neurons exist in a more readily accessible chromatin state than those in astrocytes or fibroblasts^15^. This could also account for the selective activation of inhibitory but not excitatory neuronal genes. Whether the relatively de-repressed state of inhibitory neuron genes in OPCs is mediated by Ascl1 or other transcription factors is currently unknown.

We previously showed that conditional knock-out of Olig2 in postnatal cortical OPCs converts their fate into protoplasmic astrocytes but not into neurons^58,59^. A similar fate conversion to astrocytes but not neurons occurs after deletion of HDAC3, which causes downregulation of Olig2^60^. By contrast, we did not observe astrocyte differentiation from Dlx2-transfected OPCs, despite an early loss of Olig2 (Fig. 1, Supplemental Fig. 1B). Although the exact mechanism underlying the fate choice of OPCs after loss of Olig2 remains unclear, it is likely that loss of Olig2 in the presence of Dlx2 triggers a transcription factor network that represses STAT3 and other astrocyte genes.

In OPCs transfected with Dlx2, there was a gradual loss of oligodendrocyte lineage antigens from 2 to 7 dpt, but roughly 25% still contained detectable Olig2, PDGFRα, and Sox10 at 7 dpt, suggesting that roughly 25% of OPCs are resistant to reprogramming by Dlx2 or take longer to be reprogrammed. The shutdown of oligodendrocyte lineage proteins could take longer in some OPC that are more "mature" and farther along the oligodendrocyte lineage than those that had newly differentiated from NPCs. This is supported by our recent finding that upon Olig2 deletion in OPCs, Sox10 expression disappears within a few days when Olig2 is deleted prenatally but remains for several weeks when deleted at P18^58,59^.

### Partial recapitulation of developmental transcription network triggered by Dlx2 misexpression in OPCs

During interneuron development in the medial ganglionic eminence, Dlx1/2 first specifies GABAergic lineages by repressing Olig2. Subsequently, in postmitotic cells, Dlx1/2 promotes further neuronal differentiation by inducing Gad1/2 and Dlx5/6 (reviewed in^61^), as well as Sp9. These immediate targets of Dlx1/2, as well as Dlx1, were all upregulated in OPCs after less than 48 h of exposure to Dlx2. We further used qPCR to examine the extent to which these secondary transcription factors were sustained in Dlx2-transfected OPCs. In our qPCR analysis, we could not attain the sensitivity obtained by RNA-seq at 2 dpt, since the OPC-derived neurons were too fragile for FACS purification at later time points, and many untransfected cells were mixed in the analyzed population. Nevertheless, the results from qPCR showed a trend for an early upregulation of Dlx1, though not statistically significant, and a significant upregulation of Dlx6 and 5, as well as significantly elevated and sustained expression of Gad1 through 14 dpt. Elevation of Dlx1 and Dlx6 was transient and declined by 14 dpt, whereas the most significant upregulation of Dlx5 expression occurred between 7 and 14 dpt.

Interestingly, two lncRNAs in the Dlx family of genes, Dlx6as and Dlx1as, were significantly upregulated at 2 dpt in our RNA-seq data, and this was further validated by qPCR, suggesting a greater magnitude of elevation of these genes compared to the other Dlx genes. The upregulation of both Dlx lncRNA transcripts was transient, and by 7 dpt they returned to a level that was not significantly above baseline levels. The steep early upregulation of the two Dlx lncRNA species could play a significant role in modulating the temporal profiles of the levels of Dlx1/5/6 genes. The specific mechanism of action of Dlx1as and Dlx6as in this context remains unclear, and reports of studies in different cell types vary. Dlx6as has been shown to recruit and form a complex with Dlx2 to activate the Dlx5/6 intergenic enhancer^62^. Thus, it is possible that the elevated expression of Dlx6as is contributing to the elevated expression of Dlx 6 and 5 observed at 7 and 14 dpt, respectively. This is consistent with the report that knockdown of Dlx1as in adult SVZ cells decreased Dlx1 and Dlx2 expression^47^. On the contrary, Dlx1as knock-out mice have higher Dlx1 expression and exhibit the phenotype of gain-of-function of Dlx1^46^. If Dlx1as positively affects Dlx1 expression in OPC-derived neurons, the early induction of Dlx1as may have played a role in inducing Dlx1 expression, but the transient nature of Dlx1as expression may not have been sufficient to sustain Dlx1 expression.

### Incomplete manifestation of interneuron class phenotypes in OPC-derived neurons

Dlx2-transfected OPCs did not exhibit the fully differentiated phenotype of any of the three major interneuron classes known to exist in the neocortex. RNA-seq data at 2 dpt did not detect upregulation of Pvalb, Sst, or Vip. By 14 dpt, we detected a small but significant increase in Sst transcript by qPCR, suggesting an incomplete differentiation toward SST + interneurons. Interestingly, Sp9, which was found to be elevated at 2 dpt by RNA-seq, is known to promote the activation of Satb1 and St18^63^, both of which were elevated at 14 dpt in qPCR analyses. Satb1 is critical for the survival and maturation of SST + and some PV + interneurons^52,53^, and is characteristically expressed as one of the signature transcripts of SST + interneurons (database by the Linnarsson lab, http://mousebrain.org/celltypes/).

During development, Dlx1 is expressed transiently in early PV + interneuron-fated progenitors and is downregulated upon PV specification, whereas Dlx1 expression is sustained as progenitor cells mature into SST + interneurons^64^. The decline of Dlx1 beyond 7 dpt in OPC-derived neurons could be related to the emergence of a transcriptional signature of SST + neurons. However, electrophysiological recordings of OPC-derived neurons revealed that 3/15 cells exhibited a train of action potentials with an average firing rate of ~ 85 Hz upon current injection with no sign of adaptation. The range of action potential firing frequency was higher than that of the fastest firing SST + neurons, but lower than that of mature PV + neurons and more similar to immature PV + neurons seen during the first postnatal week^65–67^. The most common action potential firing pattern seen among Dlx2-transfected cells was several spikes of decaying amplitude after current injection followed by inactivity. This is often seen in reprogrammed neurons^12,55,68^, suggesting a partially differentiated phenotype, possibly reflected by the density and type of Na^+^ and K^+^ channels. OPCs in different functional states have different Na^+^ channel densities, though they are generally too low to generate regenerative action potentials^69,70^. Thus, types 2 and 3 firing patterns observed in Fig. 4 likely represent a gradient of neuronal differentiation from OPCs.

The health of the OPC-derived neurons began to deteriorate beyond 21 dpt under the culture conditions used, and we were unable to examine whether the cells developed more characteristic neurochemical and biophysical phenotypes of more mature interneuron classes over time. Since progenitor cells in the medial ganglionic eminence acquire competence to produce SST + neurons earlier than PV + class of interneurons^61^, it is possible that the detection of Sst, Satb1, and St18 is reflecting the relatively early stage of interneuron maturation. The lack of detectable PV or SST immunoreactivity in OPC-derived neurons at 14 dpt is also consistent with their immature state. We have also noted that Lhx6, a critical secondary interneuron transcription factor, was not upregulated in Dlx2-transfected OPCs. Additional transcription factors such as Nkx2-1 may be needed in parallel with the transcriptional network that is activated by Dlx2.

Of note, our electrophysiological recordings were conducted from OPC-derived neurons that had integrated Dlx2-IRES2-mCherry into the genome. While beneficial for lineage tracing of transfected cells, sustained Dlx2 expression may have impeded full neuronal maturation. Future studies may be conducted with temporal control of gene Dlx2 expression^71^ to further promote maturation of OPC-derived interneurons.

Inputs from surrounding neurons, particularly those from excitatory neurons promote the survival of postmitotic immature interneurons^72–74^. Thus, it is possible that further maturation of OPC-derived neurons in a recent in vivo study in which striatal OPCs were reprogrammed into neurons with multiple transcription factors^14^ may have been facilitated by the local in vivo environment. Our findings provide a logical framework for further testing strategies to promote interneuron reprogramming from OPCs under pathological conditions in which greater inhibitory neuron function is needed.

## Supplementary Information

Supplementary Information 1.

Supplementary Information 2.

Supplementary Information 3.

Supplementary Information 4.

Supplementary Information 5.

Supplementary Information 6.

## References

[1] Dawson MR, Polito A, Levine JM, Reynolds R (2003). NG2-expressing glial progenitor cells: An abundant and widespread population of cycling cells in the adult rat CNS. Mol. Cell Neurosci..

[2] Nishiyama A, Komitova M, Suzuki R, Zhu X (2009). Polydendrocytes (NG2 cells): Multifunctional cells with lineage plasticity. Nat. Rev. Neurosci..

[3] Nishiyama A, Boshans L, Goncalves CM, Wegrzyn J, Patel KD (2016). Lineage, fate, and fate potential of NG2-glia. Brain Res..

[4] Bergles DE, Richardson WD (2015). Oligodendrocyte development and plasticity. Cold Spring Harb. Perspect. Biol..

[5] Spassky N (1998). Multiple restricted origin of oligodendrocytes. J. Neurosci..

[6] Nery S, Wichterle H, Fishell G (2001). Sonic hedgehog contributes to oligodendrocyte specification in the mammalian forebrain. Development.

[7] Wichterle H, Turnbull DH, Nery S, Fishell G, Alvarez-Buylla A (2001). In utero fate mapping reveals distinct migratory pathways and fates of neurons born in the mammalian basal forebrain. Development.

[8] Kessaris N (2006). Competing waves of oligodendrocytes in the forebrain and postnatal elimination of an embryonic lineage. Nat. Neurosci..

[9] Miyoshi G, Butt SJ, Takebayashi H, Fishell G (2007). Physiologically distinct temporal cohorts of cortical interneurons arise from telencephalic Olig2-expressing precursors. J. Neurosci..

[10] Petryniak MA, Potter GB, Rowitch DH, Rubenstein JL (2007). Dlx1 and Dlx2 control neuronal versus oligodendroglial cell fate acquisition in the developing forebrain. Neuron.

[11] Silbereis JC (2014). Olig1 function is required to repress dlx1/2 and interneuron production in mammalian brain. Neuron.

[12] Heinrich C (2014). Sox2-mediated conversion of NG2 glia into induced neurons in the injured adult cerebral cortex. Stem Cell Rep..

[13] Guo Z (2014). In vivo direct reprogramming of reactive glial cells into functional neurons after brain injury and in an Alzheimer's disease model. Cell Stem Cell.

[14] Pereira M (2017). Direct reprogramming of resident NG2 glia into neurons with properties of fast-spiking parvalbumin-containing interneurons. Stem Cell Rep..

[15] Boshans, L. L. *et al.* The chromatin environment around interneuron genes in oligodendrocyte precursor cells and their potential for interneuron reprogramming. *Front. Neurosci.* (2019). 10.3389/fnins.2019.00829 PMC6694778 31440130

[16] Zhu X, Bergles DE, Nishiyama A (2008). NG2 cells generate both oligodendrocytes and gray matter astrocytes. Development.

[17] Sherafat A, Pfeiffer F, Reiss AM, Wood WM, Nishiyama A (2008). Microglial Neuropilin-1 trans-regulates oligodendrocyte expansion during development and remyelination. BioRxiv.

[18] Tamamaki N (2003). Green fluorescent protein expression and colocalization with calretinin, parvalbumin, and somatostatin in the GAD67-GFP knock-in mouse. J. Comp. Neurol..

[19] Emery B, Dugas JC (2013). Purification of oligodendrocyte lineage cells from mouse cortices by immunopanning. Cold Spring Harb. Protoc..

[20] Sommer I, Schachner M (1981). Monoclonal antibodies (O1 and O4) to oligodendrocyte surfaces: An immunocytological study in the central nervous system. Dev. Biol..

[21] Kaech S, Banker G (2006). Culturing hippocampal neurons. Nat. Protoc..

[22] Urasaki A, Morvan G, Kawakami K (2006). Functional dissection of the Tol2 transposable element identified the minimal cis-sequence and a highly repetitive sequence in the subterminal region essential for transposition. Genetics.

[23] Gotoh H (2018). NG2 expression in NG2 glia is regulated by binding of SoxE and bHLH transcription factors to a Cspg4 intronic enhancer. Glia.

[24] Yu G, Wang LG, Han Y, He QY (2012). clusterProfiler: An R package for comparing biological themes among gene clusters. Omics.

[25] Boyle EI (2004). GO::TermFinder-open source software for accessing Gene Ontology information and finding significantly enriched Gene Ontology terms associated with a list of genes. Bioinformatics.

[26] Komitova M, Zhu X, Serwanski DR, Nishiyama A (2009). NG2 cells are distinct from neurogenic cells in the postnatal mouse subventricular zone. J. Comp. Neurol..

[27] Suzuki-Hirano A, Shimogori T (2009). The role of Fgf8 in telencephalic and diencephalic patterning. Semin. Cell Dev. Biol..

[28] Swiss VA (2011). Identification of a gene regulatory network necessary for the initiation of oligodendrocyte differentiation. PLoS ONE.

[29] Marie C (2018). Oligodendrocyte precursor survival and differentiation requires chromatin remodeling by Chd7 and Chd8. Proc. Natl. Acad. Sci. U S A.

[30] Dobashi Y, Shoji M, Kitagawa M, Noguchi T, Kameya T (2000). Simultaneous suppression of cdc2 and cdk2 activities induces neuronal differentiation of PC12 cells. J. Biol. Chem..

[31] Calegari F, Huttner WB (2003). An inhibition of cyclin-dependent kinases that lengthens, but does not arrest, neuroepithelial cell cycle induces premature neurogenesis. J. Cell Sci..

[32] Feldblum S, Erlander MG, Tobin AJ (1993). Different distributions of GAD65 and GAD67 mRNAs suggest that the two glutamate decarboxylases play distinctive functional roles. J. Neurosci. Res..

[33] Pinal CS, Tobin AJ (1998). Uniqueness and redundancy in GABA production. Perspect. Dev. Neurobiol..

[34] Xu X, Roby KD, Callaway EM (2006). Mouse cortical inhibitory neuron type that coexpresses somatostatin and calretinin. J. Comp. Neurol..

[35] Fremeau RT (2001). The expression of vesicular glutamate transporters defines two classes of excitatory synapse. Neuron.

[36] Zeisel, A. *et al.* Brain structure. Cell types in the mouse cortex and hippocampus revealed by single-cell RNA-seq. *Science* **347**, 1138–1142, 10.1126/science.aaa1934 (2015). 10.1126/science.aaa1934 25700174

[37] Joshi P (2013). The functional interactome landscape of the human histone deacetylase family. Mol. Syst. Biol..

[38] Sher F, Boddeke E, Olah M, Copray S (2012). Dynamic changes in Ezh2 gene occupancy underlie its involvement in neural stem cell self-renewal and differentiation towards oligodendrocytes. PLoS ONE.

[39] Liu J (2015). Chromatin landscape defined by repressive histone methylation during oligodendrocyte differentiation. J. Neurosci..

[40] Lin H (2017). KDM3A-mediated demethylation of histone H3 lysine 9 facilitates the chromatin binding of Neurog2 during neurogenesis. Development.

[41] Huang C (2010). The dual histone demethylase KDM7A promotes neural induction in early chick embryos. Dev. Dyn..

[42] Nitarska J (2016). A functional switch of NuRD chromatin remodeling complex subunits regulates mouse cortical development. Cell Rep..

[43] Moyon S (2016). Functional characterization of DNA methylation in the oligodendrocyte lineage. Cell Rep..

[44] Zhou QP (2004). Identification of a direct Dlx homeodomain target in the developing mouse forebrain and retina by optimization of chromatin immunoprecipitation. Nucleic Acids Res..

[45] Poitras L, Ghanem N, Hatch G, Ekker M (2007). The proneural determinant MASH1 regulates forebrain Dlx1/2 expression through the I12b intergenic enhancer. Development.

[46] Kraus P (2013). Making sense of Dlx1 antisense RNA. Dev. Biol..

[47] Ramos AD (2013). Integration of genome-wide approaches identifies lncRNAs of adult neural stem cells and their progeny in vivo. Cell Stem Cell.

[48] Berghoff EG (2013). Evf2 (Dlx6as) lncRNA regulates ultraconserved enhancer methylation and the differential transcriptional control of adjacent genes. Development.

[49] Tasic B (2016). Adult mouse cortical cell taxonomy revealed by single cell transcriptomics. Nat. Neurosci..

[50] Mayer C (2018). Developmental diversification of cortical inhibitory interneurons. Nature.

[51] Mi D (2018). Early emergence of cortical interneuron diversity in the mouse embryo. Science.

[52] Close J (2012). Satb1 is an activity-modulated transcription factor required for the terminal differentiation and connectivity of medial ganglionic eminence-derived cortical interneurons. J. Neurosci..

[53] Denaxa M (2012). Maturation-promoting activity of SATB1 in MGE-derived cortical interneurons. Cell Rep..

[54] Wapinski OL (2017). Rapid chromatin switch in the direct reprogramming of fibroblasts to neurons. Cell Rep..

[55] Heinrich C (2010). Directing astroglia from the cerebral cortex into subtype specific functional neurons. PLoS Biol..

[56] Zhang Y (2014). An RNA-sequencing transcriptome and splicing database of glia, neurons, and vascular cells of the cerebral cortex. J. Neurosci..

[57] Parras CM (2007). The proneural gene Mash1 specifies an early population of telencephalic oligodendrocytes. J. Neurosci..

[58] Zhu X (2012). Olig2-dependent developmental fate switch of NG2 cells. Development.

[59] Zuo H (2018). Age-dependent decline in fate switch from NG2 cells to astrocytes after Olig2 deletion. J. Neurosci..

[60] Zhang L (2016). Hdac3 interaction with p300 histone acetyltransferase regulates the oligodendrocyte and astrocyte lineage fate switch. Dev. Cell.

[61] Lim L, Mi D, Llorca A, Marin O (2018). Development and functional diversification of cortical interneurons. Neuron.

[62] Feng J (2006). The Evf-2 noncoding RNA is transcribed from the Dlx-5/6 ultraconserved region and functions as a Dlx-2 transcriptional coactivator. Genes Dev..

[63] Liu Z (2019). Sp9 regulates medial ganglionic eminence-derived cortical interneuron development. Cereb. Cortex.

[64] Hu JS, Vogt D, Sandberg M, Rubenstein JL (2017). Cortical interneuron development: A tale of time and space. Development.

[65] Pan G, Yang JM, Hu XY, Li XM (2016). Postnatal development of the electrophysiological properties of somatostatin interneurons in the anterior cingulate cortex of mice. Sci. Rep..

[66] Wamsley B, Fishell G (2017). Genetic and activity-dependent mechanisms underlying interneuron diversity. Nat. Rev. Neurosci..

[67] Tremblay R, Lee S, Rudy B (2016). Gabaergic interneurons in the neocortex: From cellular properties to circuits. Neuron.

[68] Vierbuchen T (2010). Direct conversion of fibroblasts to functional neurons by defined factors. Nature.

[69] Clarke LE (2012). Properties and fate of oligodendrocyte progenitor cells in the corpus callosum, motor cortex, and piriform cortex of the mouse. J. Neurosci..

[70] Spitzer, S. O. *et al.* Oligodendrocyte progenitor cells become regionally diverse and heterogeneous with age. *Neuron* **101**, 459–471 e455, 10.1016/j.neuron.2018.12.020 (2019). 10.1016/j.neuron.2018.12.020 PMC6372724 30654924

[71] Yang N (2017). Generation of pure GABAergic neurons by transcription factor programming. Nat. Methods.

[72] Corner MA, Ramakers GJ (1992). Spontaneous firing as an epigenetic factor in brain development–physiological consequences of chronic tetrodotoxin and picrotoxin exposure on cultured rat neocortex neurons. Brain Res. Dev. Brain Res..

[73] Katz LC, Shatz CJ (1996). Synaptic activity and the construction of cortical circuits. Science.

[74] Takada N, Yanagawa Y, Komatsu Y (2005). Activity-dependent maturation of excitatory synaptic connections in solitary neuron cultures of mouse neocortex. Eur. J. Neurosci..

