# Supplementary figures and images for: Direct reprogramming of oligodendrocyte precursor cells into GABAergic inhibitory neurons by a single homeodomain transcription factor Dlx2
Source: Sci Rep. 2021 Feb 11;11:3552. doi: 10.1038/s41598-021-82931-9 (PMC7878775; doi:10.1038/s41598-021-82931-9)

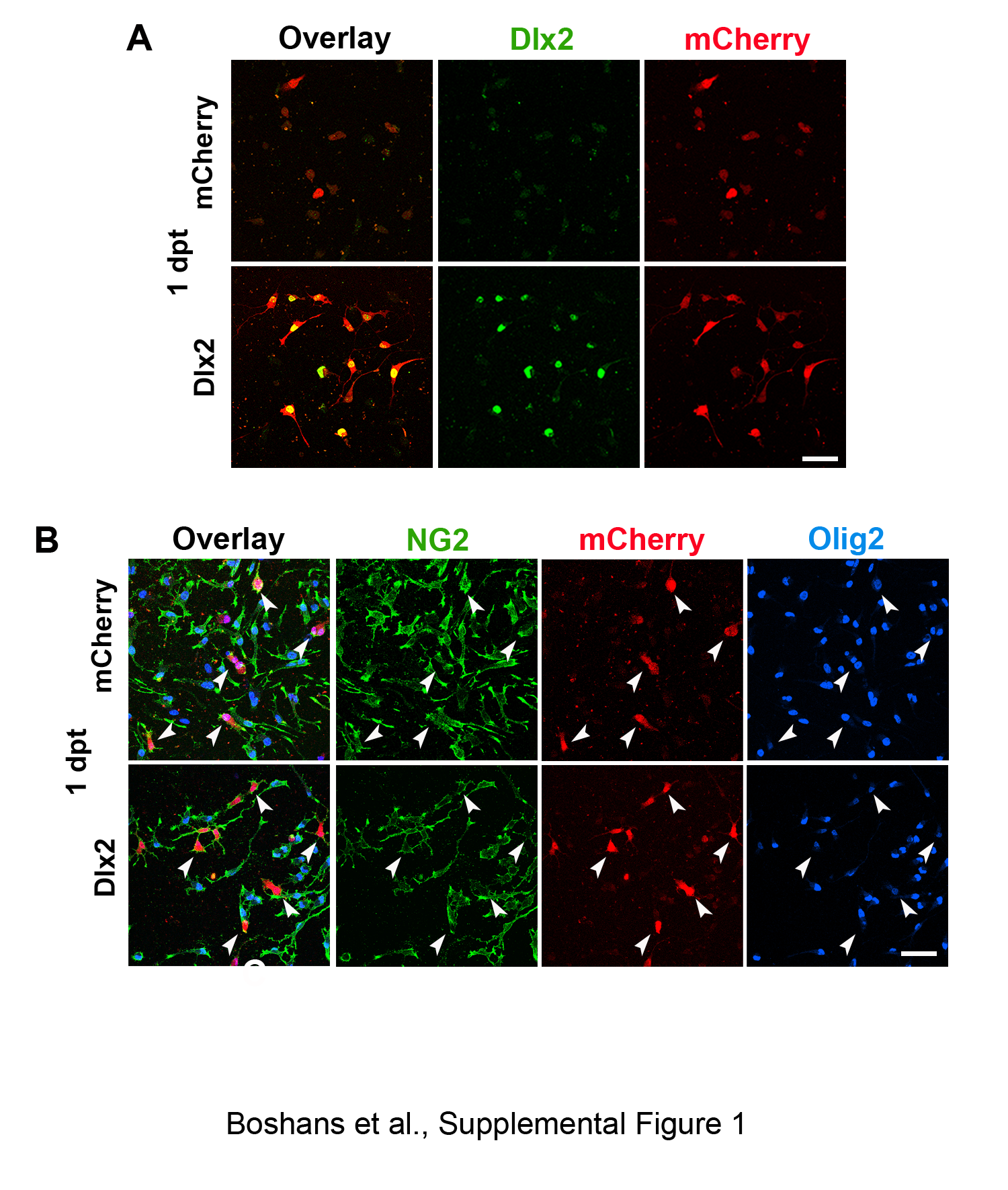

Supplement: Supplementary file 2 — Supplementary Information 2. [file 41598_2021_82931_MOESM2_ESM.tif]

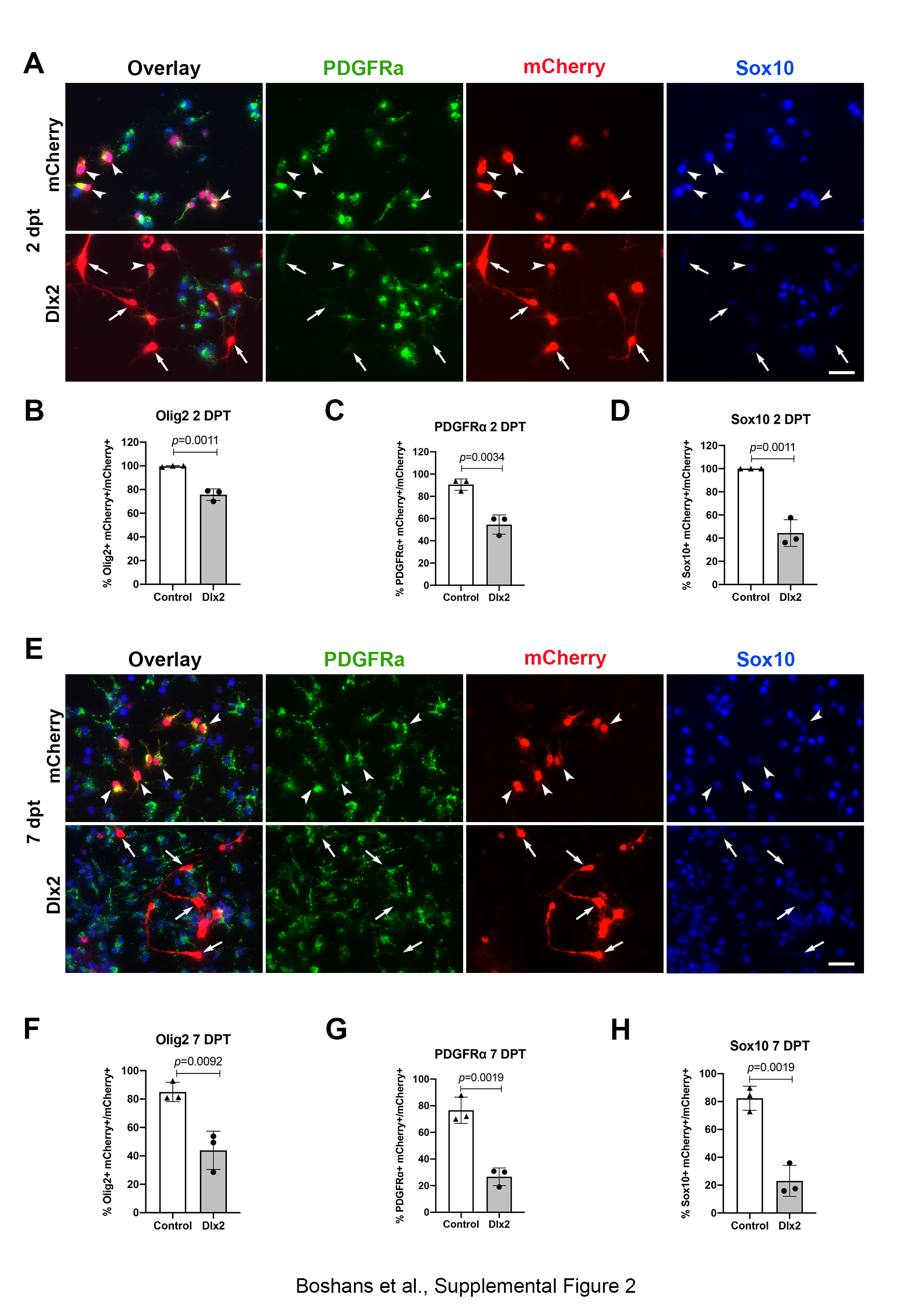

Supplement: Supplementary file 3 — Supplementary Information 3. [file 41598_2021_82931_MOESM3_ESM.tif]

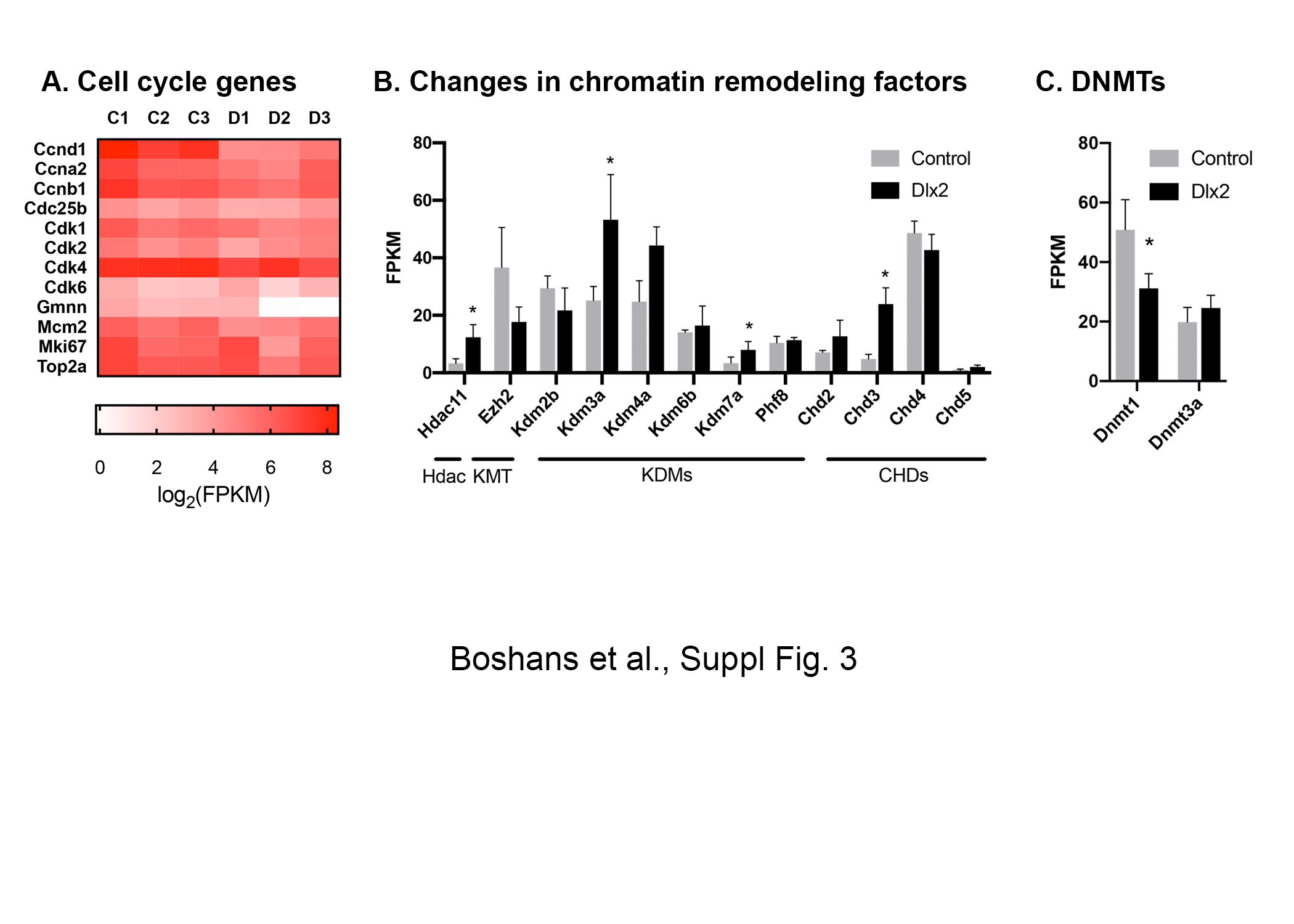

Supplement: Supplementary file 4 — Supplementary Information 4. [file 41598_2021_82931_MOESM4_ESM.tif]

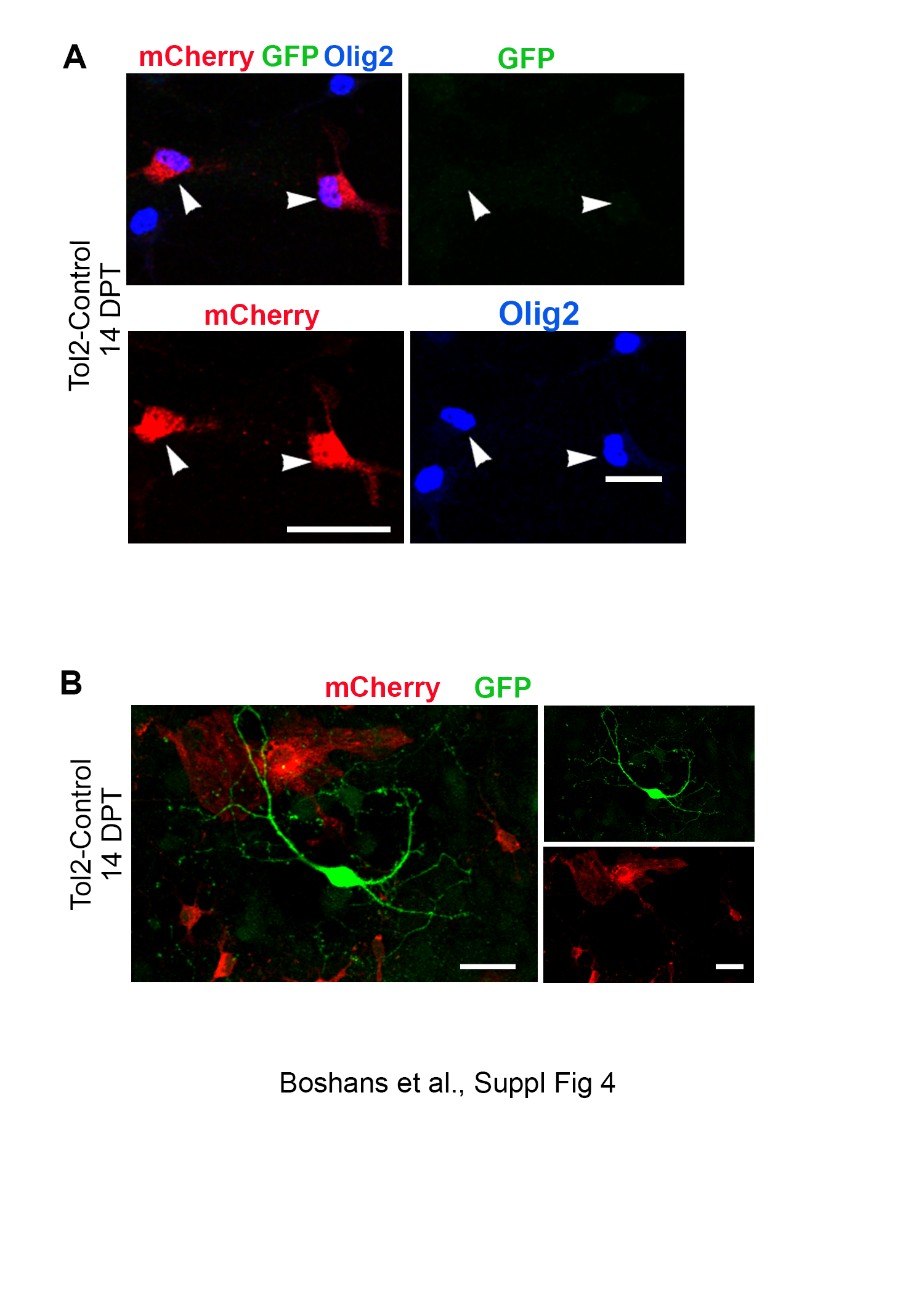

Supplement: Supplementary file 5 — Supplementary Information 5. [file 41598_2021_82931_MOESM5_ESM.tif]
