# Supplementary material for: Direct reprogramming of oligodendrocyte precursor cells into GABAergic inhibitory neurons by a single homeodomain transcription factor Dlx2
Source: Sci Rep. 2021 Feb 11;11:3552. doi: 10.1038/s41598-021-82931-9 (PMC7878775; doi:10.1038/s41598-021-82931-9)
Supplement: Supplementary file 6 — Supplementary Information 6. [file 41598_2021_82931_MOESM6_ESM.docx]

Supplemental materials

**Supplemental Table 1.** List of significantly differentially expressed genes in Dlx2-transfected OPCs (*p*-adj<0.01) ordered from highest to lowest *p*-adj value.

**Supplemental Figure 1**. Dlx2-transfected cells are OPCs and express Dlx2 protein.

**Supplemental Figure 2.** Dlx2-transfected cells downregulate oligodendrocyte lineage proteins.

**Supplemental Figure 3.** Gene expression changes of cell cycle regulators and chromatin modifiers revealed by RNA-seq at 2 dpt.

**Supplemental Figure 4**. Control-transfected cells stay within the oligodendrocyte lineage.
